# Surgical Amputation of the Digit: An Investigation Into the Technical Variations Among Hand Surgeons

**Published:** 2013-02-28

**Authors:** Andrew Li, Matthew Meunier, Hans-Oliver Rennekampff, Mayer Tenenhaus

**Affiliations:** ^a^Department of General Surgery, Harbor UCLA Medical Center, Torrance, CA; ^b^Department of Orthopedic Surgery, UC San Diego Health System, La Jolla, CA; ^c^Department for Plastic, Hand and Reconstructive Surgery, Medical School, Hannover, Germany; ^d^Department of Plastic and Reconstructive Surgery, UC San Diego Health System, La Jolla, CA

## Abstract

**Objective:** Digital injuries are common and frequently complicate occupational hazards and trauma. The management of these injuries often necessitates digital amputation, and a variety of different amputation techniques are advocated and employed by hand surgeons. In this survey study, we investigate the variation in technical detail among a group of hand surgeons when performing digital amputations, specifically the preferred management of the residual articular cartilage, transected nerves, and phalangeal contouring. **Methods:** We reviewed the literature on techniques in digital amputation and created a 7-question survey that targeted controversial issues within this specific topic. We then sent this survey electronically to the members of the American Society for Surgery of the Hand and reviewed the responses of the respondents (n = 592, 20%). **Results:** There was a mixed response regarding whether or not to remove the articular cartilage when disarticulating, nearly a 50% split between the respondents. Most would perform a “pull and resect” technique for transected nerves. Phalangeal contouring was generally agreed upon, though the technique in doing so varied from performing condylectomies, to bony contouring only, to some combination of both. **Conclusions:** We detected a substantial variation in technique among our group of hand surgeons regarding the treatment of articular cartilage and the method of phalangeal contouring. There was more consensus regarding the treatment of transected nerve. It is interesting that to date, the aforementioned issues in digital amputation have not been critically evaluated by definitive and well-controlled studies.

Digital injuries, such as fingertip injuries, can be devastating for people of all ages, and in adults these types of injuries are commonly associated with occupational hazards.^1^ Mechanisms of injury include lacerations, crush injuries, as well as avulsions, in order of decreasing frequency.^1^ For the amputated or dysvascular digit, there are 2 general treatment options: revascularization or completion of amputation. The indications for digital replantation are not rigidly defined but are typically undertaken in cases where the thumb or multiple fingers are amputated and in pediatric cases. In these specific situations, replantation may facilitate an improved functional outcome despite the fact that the reattached digits usually do not attain their original level of function.^1^^-^3 In most other situations, single digit replantation frequently results in a stiff, minimally sensate digit that may hamper the movement of the neighboring digits, globally disrupting hand function.^4^ In these situations, a digital amputation is generally considered a preferable treatment option in our realm of practice within the United States.

When approaching upper extremity amputations, it is important to preserve functional length, provide durable and supple coverage, preserve useful sensibility, prevent painful neuromas and adjacent joint contractures, and expedite the patient's ability to return to work.^4^ Other important goals include salvaging all remaining digits and digital remnants, preserving the palm, and providing a functional thumb and opposing digit for pinch and grasp functions.^5^^-^7 Wilhelmi^4^ summarizes these goals as “providing a mobile, stable, painless stump with the least interference from the remaining tendon and joint function to provide the most useful amputation stump.” Although the goals of surgery in these situations are more or less congruent among hand surgeons, there is a significant amount of variation in technical detail when different surgeons perform digital amputations.

The purpose of our study is to determine whether there is consensus with regard to the preferred management of the residual articular cartilage, transected nerves, and phalangeal contouring when performing digital amputations.

## MATERIALS AND METHODS

A 7-question surgery was created that asked questions concerning certain issues when performing digital amputations. These issues were targeted because our literature search demonstrated a significant amount of variation when different surgeons addressed each issue. The survey questions are as follows:

1. In performing noninfected traumatic digital amputations at the level of the proximal or distal interphalangeal (IP) joints, which do you prefer?
  - Disarticulation
  - Resection through bone
  - Other (describe)
2. If you resect through bone, which is the most applicable technique?
  - Smoothing all bone edges
  - It is more important to smooth the dorsal edge than the volar edge
  - Other (describe)
3. With respect to IP joint disarticulations, do you reduce the size of the remnant phalangeal heads?
  - No
  - Yes
4. If your response to Q3 was “yes,” then by which of the following methods?
  - By trimming the condyles
  - By contouring the phalangeal head
  - Other (describe)
5. With respect to IP joint disarticulations, do you remove the articular cartilage?
  - No
  - Yes
6. Which is most applicable with regard to the digital nerves when you perform a digital amputation?
  - I do not resect nerve
  - I pull and resect nerve
  - Other (describe)
7. Which of the following do you consider the important in preventing neuroma formation?
  - Placing nerve end away from anticipated area of repeated trauma
  - Placing nerve end away from incision site
  - Placing nerve end away from digital blood vessel
  - Other (describe)

The survey was distributed electronically among members of the American Society for Surgery of the Hand, a society that consists of hand surgeons who have passed the CAQSH (Certificate of Added Qualifications in Surgery of the Hand) or SOTH (Surgery of the Hand) as administered by the appropriate Specialty Board (ABOS, ABPS, or ABS). A total of 592 participated in the survey out of 2970 members who were contacted, about a 20% response rate.

## RESULTS

**Figure 1:**
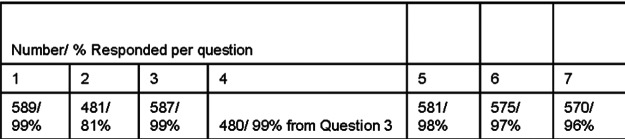
Number and percent response per question.

Following the distribution of the survey, we tabulated our results in the figures appearing later. Figure 1 illustrates the number of respondents per question and the overall response rate to each.

### Disarticulation and articular cartilage

**Figure 2:**
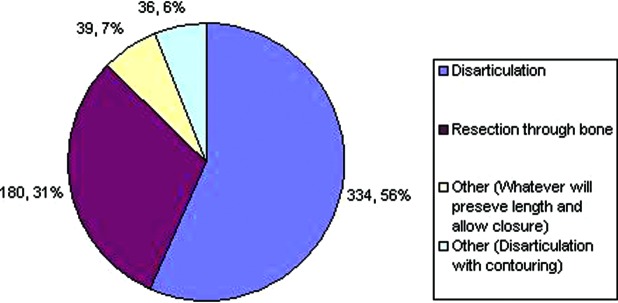
Preference regarding disarticulation versus resection through bone.

**Figure 3:**
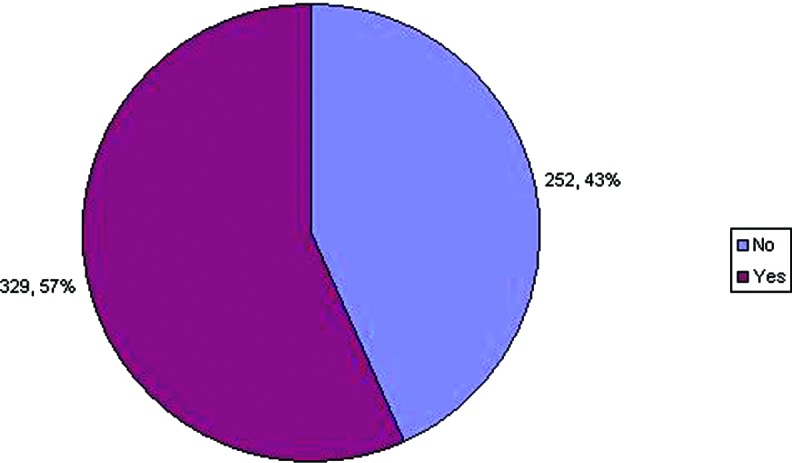
Preference regarding smoothing bony edges following resection.

In the case of disarticulation versus resection through bone when performing digital amputations, 56% of respondents disarticulate, while 31% prefer to resect through bone (Fig 2). Others responded that they would do a combination of both, specifically disarticulation in conjunction with some form of resection, that is, contouring either with condylectomy or shaving/removing the cartilage. Others responded that they would perform either technique depending on whichever would allow the most preservation of length and allow closure. With regard to removing cartilage when performing disarticulations, 329 (57%) responded that they would remove articular cartilage, while 252 (43%) said they would not (Fig 3).

### Smoothing bone following resection

**Figure 4:**
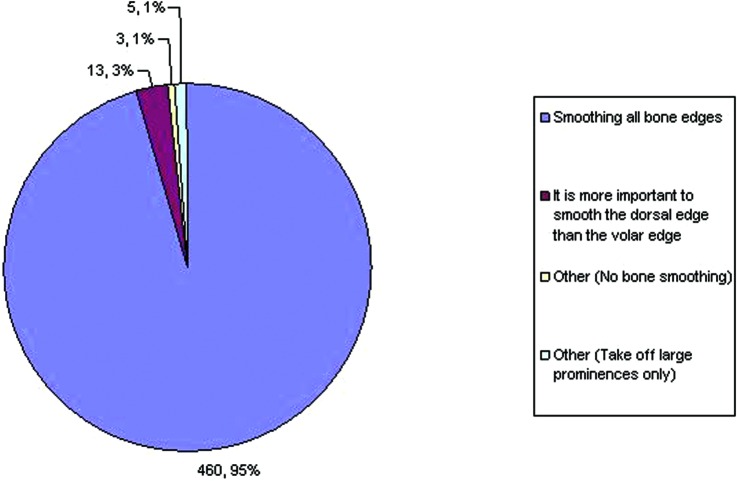
Preference regarding phalangeal heads following disarticulation.

We found that about 99% of respondents smooth the bone in some form or another following any kind of resection through bone (Fig 4). Only about 1% (total of 3) of the respondents felt that no bone smoothing was needed. Of those who agreed on bone smoothing, the majority (96%) felt that smoothing all bone edges was important, while some of them felt that it was sufficient either to smooth only the large prominences or that the dorsal edge was more important to smooth than the volar edge.

### Phalangeal contouring

**Figure 5:**
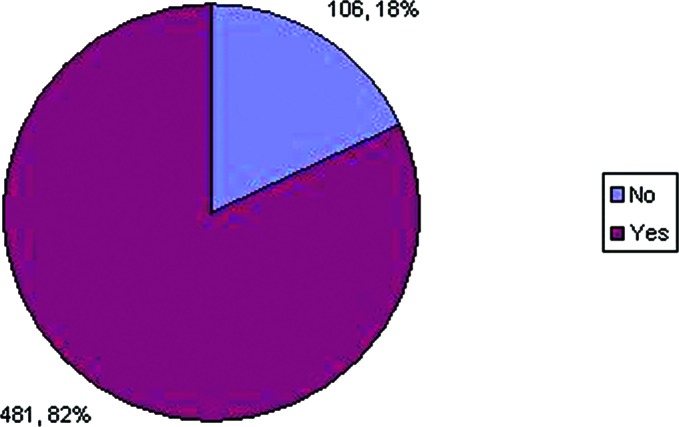
Preference regarding the method by which the size of phalangeal heads is reduced.

**Figure 6:**
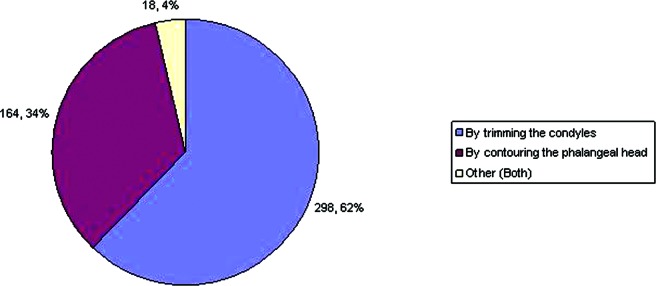
Preference regarding the management of articular cartilage

A total of 481 (82%) respondents said that they would reduce the size of the phalangeal heads (Fig 5). However, the technique that the respondents used to do so varied (Fig 6). We found that about two thirds of respondents would perform condylectomy to reduce the phalangeal head. About one third would just contour the head without condylectomy, and about 4% would choose a combination of both

### Transected nerve

**Figure 7:**
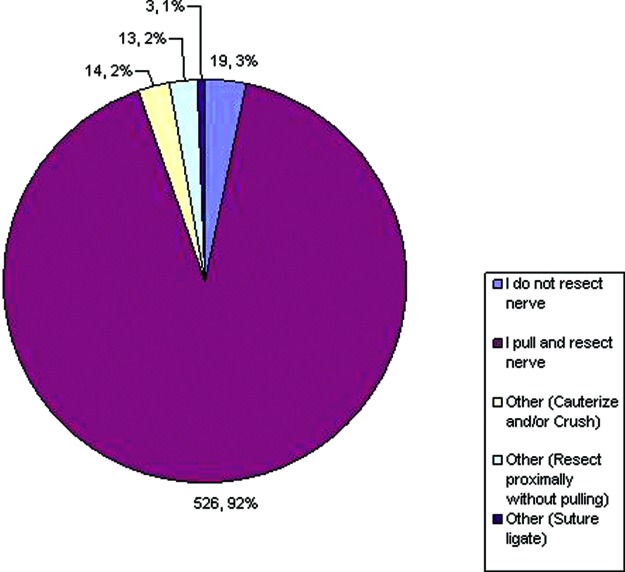
Preference regarding the management of digital nerves.

**Figure 8:**
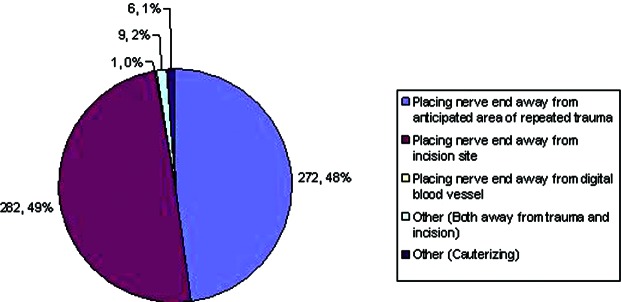
Important factors in preventing neuroma formation.

Regarding the treatment of digital nerves during digital amputations (Fig 7), 556 (97%) perform some form of digital nerve resection, while only 19 (3%) responded that they do not resect nerve. Of those that do resect, the majority pull and resect, while others try not to apply any traction while resecting. Cautery and/ or crushing the nerve along with suture ligation of the nerve were other mentioned techniques. Interestingly, the topic regarding neuroma formation created some differences, about half responding that placing the nerve away from areas of trauma was key in prevention, the other half saying that placing the nerve away from the incision was more important (Fig 8).

## DISCUSSION

There was a satisfactory response to each question (see Fig 1). Of note is that the percentage of respondents to question 2 was less than those of the other questions. A possible explanation for this is the wording of the question in that those who did not respond perhaps thought that the question was to be answered depending on his/her response to the first question. When we designed this question, however, we intended it to be answered independently of the first question. Also of note is that the percentage of respondents to question 4 was calculated on the basis of those who answered “yes” in question 3.

Following our perusal of the National Center for Biotechnology Information database using the search terms “surgical amputation” and “digit” and analysis of our study results, we found there to be some consensus on certain technical aspects of digital amputations, while in other aspects, there was a greater degree of variation. This variety may lend itself to the diversity of tissues found in the hand, including bone, tendon, nail, cartilage, vascular structures, and nerves.

In dealing with the bony injuries, initial treatment should include the removal of bone chips and devitalized bone.^4^ Following this, there is discussion regarding resection of bone versus disarticulation. In the case of disarticulations of the IP joints, some surgeons describe the remnant bulbous phalangeal heads as cosmetically unappealing and functionally cumbersome.^8^ These surgeons advocate trimming the condyles^4^^,^^9^ and achieving a smooth cone-shaped stump,^10^ which is consistent with our findings in question 3 that show a majority of hand surgeons would reduce the size of these phalangeal heads. However, our study demonstrated that the methods of phalangeal contouring varied from performing condylectomy, to contouring bone only, to a combination of both.

When bone is transected during a digital amputation, it is generally agreed upon that the edges of the bony stump ought to be smoothed out,^4^^,^^9^ and the stump contoured appropriately.^8^ Some authors describe the greater importance of smoothing out the dorsum of the amputated bone in particular rather than the volar aspect, stating that dorsal skin flaps tend to be much thinner and less durable than palmar skin and soft tissue.^11^ In terms of preference of disarticulation to resection through bone, our survey results were mixed, though our respondents tended toward disarticulation. For resection through bone, we found that the majority would smooth out all bony edges and not necessarily prefer smoothing out the volar edge more so than the dorsal aspect.

The treatment of articular cartilage has generated some diversity in surgical technique. Some surgeons encourage preserving the articular cartilage, stating that it acts as a “shock pad,” reducing stump pain when compared with stumps created upon bone edges.^4^ Whitaker et al^12^ investigated this issue with a case series of 20 male patients who underwent surgical disarticulations for traumatic injuries of the fingers, involving a range of joints including metacarpophalangeal, MCP, proximal inter-phalangeal (IP), and distal IP joints. They found that finger stumps created from transarticular amputations with retained cartilage showed greater flexibility, compared to digits that were disarticulated in addition to chondrectomy. These observations were often made in patients who had received both the cartilage sparing and nonsparing disarticulations on the same hand. Other perceived benefits included preservation of stump length, as well as allowing for a simpler surgery with less intraoperative bleeding. Conolly and Goulston^10^ echo this way of thinking, stating that the articular cartilage is “physiologically designed for painless transmission of force.”^10^ In contrast, others like Thompson^9^ state that cartilage should be removed, as it may contribute to a tender stump. Our survey results echo this degree of variation found in the literature, as we found nearly a 50% split between removing and not removing the cartilage.

The greatest concern with remnant nerve tissue is the formation of painful neuromas. General adages to prevent these include keeping the end of the nerve away from any area of foreseeable trauma,^4^ keeping the nerve end away from operative incision,^13^ placing nerve ends away from stump scar,^8^ and dissecting nerve away from the digital blood vessels.^10^ Specific technical descriptions include resecting the nerve as “high up as possible”^14^ and as short as possible,^11^ allowing the nerve end to retract thereafter,^9^^,^^11^ performing bilateral traction neurectomies on each affected digit, and keeping the end of the nerve between 1 and 1.5 cm from the end of the stump.^4^ Conolly and Goulston describe cutting only the distal 0.5 cm of the nerve.^10^ From our survey results, it seems that the general preference among this group of hand surgeons is to use a “pull and resect” technique.

Some issues were not addressed with our questionnaire. These include treatment of tendons, amputations through the nail bed and amputations due to severe infection of the digit.

Tendon insertions should be preserved when possible.^4^ However, there is general consensus that transected tendons ought to be cut cleanly and allowed to retract.^4^^,^^8^^,^^9^^,^^11^ Suturing transected tendon to remnant bone should be avoided,^4^^,^^9^ as doing so may result in tethering the tendons of other digits.^9^ A good example is the case of the flexor digitorum profundus, which if sutured to bone can result in the quadriga effect where flexor function of all other digits is reduced, resulting in weaker grip.^8^ There are exceptions to the aforementioned conditions, as it may be that the injury can be remedied with a substituted motor unit.^8^ Pinch function is the main task of the index finger.^5^ However, in index finger amputation injuries that occur at or proximal to proximal IP joint, this function is preferentially taken on by the middle finger.^5^^,^^8^

When treating distal phalanx injuries, there is general consensus that the nail bed ought to be removed completely to prevent the formation of painful nail remnant nodules.^9^^-^11 Moreover, these nodules may become subsequently infected, making it important to prevent their formation.^9^

A brief mention of management of finger infections is worthwhile. Most commonly, these infections stem from traumatic injuries, with diabetes mellitus and neuropathy being significant risk factors.^15^ Management of these infections usually consists of a combination of antibiotics, immobilization, and elevation,^16^ where initial immobilization serves to prevent the spreading of the infection across fascial planes, and elevation, to prevent edema that can mask an underlying abscess and keep the hand from being placed in a contracture-preventing, physiologic position.^16^ Surgical intervention usually consists of exploration of bite wounds, adequate debridement, and incision and drainage with debridement in cases of underlying soft tissue infections, such as those involving the dorsal subcutaneous space, flexor tendon sheath, nail bed, finger pad, and joint space.^16^ More aggressive surgical debridement and excision is mandated in cases of necrotizing infections, which are associated with intravenous drug abuse, diabetes mellitus, alcoholism, immunocompromise, and the indigent.^16^ In a case series of studying hand infections in 25 diabetic patients, Kour et al^17^ performed surgery on 24 (96%) of those patients, 21 with debridement alone and 3 needing amputation, congruent with their advocacy of earnest debridement in the control of the infectious source. Vorderwinkler et al^18^ present a more recent case series investigating 40 patients with infections of the IP joints. They describe a similar approach with arthrotomy and irrigation with aggressive debridement, adding that joint preservation should only be contemplated when there is no visible damage to the articular cartilage from the infectious process. Otherwise, their recommendation is to resect the articular surface with subsequent arthrodesis and to leave the condyle to leave the corticalis closed. We echo the need to remove cartilage in infections necessitating digital amputation as it is a nonperfused tissue, making it prone to infectious destruction.

Our survey study suffered a low response rate, which may be explained by the relatively short window for response, which was about 2 weeks from the time of distribution to the final analysis of results. Had we extended this period, we may have accrued more respondents. In addition, our survey was limited to the United States as we were initially interested in this particular population given our own experiences and observations of hand surgeons in this region. However, a future survey study may address similar issues among hand surgeons in other parts of the world. Finally, our survey study cannot make any definitive clinical recommendations, something that is secondary to the nature of our study. However, we believe that it does elicit variation in technique within our surveyed population.

## CONCLUSIONS

We found that the surveyed population of hand surgeons tends to prefer disarticulation in cases of traumatic digital amputation that occur at the proximal or distal IP joint. The majority of them will then contour the remnant phalangeal head, usually with condylectomies, and about half will remove the cartilage. Should resection through bone be absolutely necessary, the consensus is to smooth all bony edges with particular attention to the dorsal cortex. The majority would then perform traction neurectomies. Irrespective of technique, the ultimate goal is a stable, minimally symptomatic digit that maximizes residual function.

It is interesting that despite the long history of performing surgical amputations of the digit, issues such as the treatment of residual articular cartilage, transected nerves, and phalangeal contouring have not been critically evaluated by definitive and well-controlled studies. Overall, this may reflect the general notion that there is no best way to perform a digital amputation.
